# Supplementary material for: A Meroterpenoid from Tibetan Medicine Induces Lung Cancer Cells Apoptosis through ROS-Mediated Inactivation of the AKT Pathway
Source: Molecules. 2023 Feb 17;28(4):1939. doi: 10.3390/molecules28041939 (PMC9963024; doi:10.3390/molecules28041939)
Supplement: Supplementary file 1 [file molecules-28-01939-s001.zip › molecules-2166529-supplementary.pdf]

Supplementary Materials

A Meroterpenoid from Tibetan Medicine Induces Lung Cancer Cells Apoptosis through ROS-Mediated Inactivation of the AKT Pathway

Yi Huang [1,2,†], Yun Huang [3,†], Ge Zhu [4], Bingzhi Zhang [2], Yujia Zhu [5], Bin Chen [6,*], Xiaoxia Gao [2,*] and Jie Yuan [1,4,*]

[1] Key Laboratory of Tropical Disease Control (Sun Yat-sen University), Ministry of Education, Guangzhou 510080, China
[2] School of Pharmacy, Guangdong Pharmaceutical University, Guangzhou 510006, China
[3] School of Basic Medical Sciences, Southern Medical University, Guangzhou 510515, China
[4] Department of Biochemistry, Zhongshan School of Medicine, Sun Yat-sen University, Guangzhou 510080, China
[5] School of Public Health, Sun Yat-sen University, Guangzhou 510080, Guangdong, China
[6] Southern Laboratory of Ocean Science and Engineering, Zhuhai 519000, China
* Correspondence: chenbin@sml-zhuhai.cn (B.C.); gaoxxia91@163.com (X.G.); yuanjie@mail.sysu.edu.cn (J.Y.)
† These authors contributed equally to this work.

Table S1. NMR Data of Compound D1399 ($CDCl_3$), Measured at 400 MHz (^1H) and 100 MHz (^{13}C).

No.	δ H (400 MHz, mult., *J* in Hz)	δ C (100 MHz)	HMBC
1	5.01(*cis*, br d, 10.3)	115.5(CH_2)	C-3
	5.11(*trans*, br d, 17.0)		C-2, C-3
2	5.93(m)	141.6(CH)	C-3, C-4, C-5, C-14, C-15, C-16,
3	2.89(m)	37.9(CH)	C-3, C-4, C-15
4	5.50(d, 6.6)	122.8(CH)	C-3, 5-Me, C-6
5		136.3(C)	
5-Me	1.77(s)	25.4(CH_3)	C-4, C-5, C-6
6	2.88(m)	52.3(CH)	C-4, C-5, C-7, 7-Me, C-12, C-13, C-14
7		47.7(C)	
7-Me	1.88(s)	23.9(CH_3)	C-6, C-7, C-8, C-12
8	1.71(m)	48.4(CH_2)	C-7, 7-Me, C-9, C-10
	0.95(m)		C-7, C-9, C-10, C-12
9	1.77(m)	28.4(CH)	C-10
9-Me	0.89(d, 6.6)	23.1(CH_3)	C-8, C-9, C-10
10	1.77(m)	44.8(CH_2)	C-8, C-9, 9-Me, C-11, 11-Me, C-12
	0.46(m)		C-8, C-9, 11-Me, C-12
11	1.88(m)	27.2(CH)	C-6, C-10,11-Me
11-Me	0.99(d, 6.5)	20.0(CH_3)	C-10, C-11, C-12
12	1.21(m)	53.6(CH)	C-6, C-7, 7-Me, C-8, C-11, 11-Me, C-13
13	4.36(dd, 6.2, 2.1)	92.1(CH)	C-7, C-12, C-15, C-24
14	2.19(m)	42.5(CH)	C-3, C-5, C-6, C-7, C-12, C-13, C-15, C-16
15	2.85(s)	51.6(CH)	C-2, C-3, C-4, C-6, C-13, C-14, C-16, C-17
16		201.9(C)	
17		142.0(C)	
18	6.53(s)	149.7(CH)	C-1′, C-16, C-17, C-19
19		92.9(C)	
19-OMe	3.22(s)	52.1(CH_3)	C-19
20	3.27(m)	44.8(CH_2)	C-18, C-19, C-21, C-22, C-26,
21		129.6(C)	
22	7.28(m)	129.9(CH)	C-19, C-20, C-24, C-26
23	7.10(dd, 8.4, 2.4)	122.4(CH)	C-21, C-24, C-25
24		158.1(C)	
25	6.92(dd, 8.4, 2.4)	125.5(CH)	C-21, C-23, C-24,
26	7.04(br d, 5.9)	131.3(CH)	C-19, C-20, C-22, C-24
1′		169.3(C)	

^1H and ^{13}C NMR spectral data for D1399, in $CDCl_3$.

Table S2. 50% growth inhibition concentration (IC_{50}, μM) of D1399 for different cancer cell lines.

Cancer	Cell Line	IC_{50} (μM)
breast cancer	MDA-MB-231	1.69 ± 0.1
breast cancer	MCF-7	2.62 ± 1.06
melanoma	MDA-MB-435	0.89 ± 0.26
liver carcinoma	HepG2	1.02 ± 2.12
colorectal carcinoma	HCT-116	5.02 ± 1.39

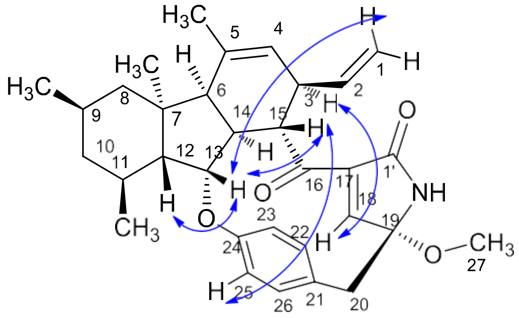

Key NOESY correlations of D1399

Figure S1. The relative configuration structure of D1399 and its key NOESY correlations.
